# First Studies in Anthropology

**Published:** 1893-12-09

**Authors:** 


### The Study of Man.

### II.—FIRST STUDIES IN ANTHROPOLOGY.

It is seldom easy to estimate the value of individual contributions to knowledge without reference, more or less explicit, to the previous state of the science which they go to expand, and to the problems which most press for solution at the time when they appear. And this is not least so in the study of man, which has advanced, perhaps more than other inquiries, through successive vigorously, and often bitterly, fought controversies, often in themselves even trifling, and only memorable for the stimulus which they gave to the accumulation of materials which have acquired a permanent importance.

Both in the classical and in the modern world men have been occupied first with the questions presented by the world outside and around them, and have only turned to the investigation of their own nature, even in the physical sense, after these earlier sciences have been formulated in some kind of order. In Greece, in particular, anthropology as such can hardly be said to have had a beginning. The views of Greek physicists on the origin and characteristics of man, of which we get glimpses in surviving fragments of Empedokles or Demokritos, and in the fine poetic summary of Lucretius, are speculations and nothing more; and only where the last-named reconstructs in detail the origins of culture and society do we find traces of genuine observation, or of a comparison of ethnographic data. It is true that travellers and historians like Herodotos, and medical writers like Hippokrates, do not wholly neglect the materials which were ready to their hand; but their *obiter dicta* have hardly a greater value as theory, than their vague and often palpably fabulous statements as evidence; and the characteristic repugnance and contempt which a Greek entertained for all foreigners as such without exception, were in themselves enough to make any systematic research in this field impossible. The elder Pliny is in fact the first of the ancients who can be said to have contemplated this kind of study in a separate light; but his inability, here as elsewhere, to digest anything out of his enormous mass of material, as well as to criticise and appreciate his authorities, leaves his collections an "unbound scrap-book" of very slight independent value.

From the side, however, of medical research, the contributions to our study are more important and systematic. Aristotle seems to have seconded his speculations on the affinity between man and the apes by direct observation, and Galen certainly dissected monkeys, though it is not clear that human anatomy was studied in this way before the close of the Middle Ages; except at Alexandria, where specially medical researches were carried on, by Erasistratos and Herophilos, at a much earlier time. The new era begins with Vesalius (1544); but, in this special regard, there is little that is noteworthy before the close of the seventeenth century.

Nor, on the ethnological side, does the discovery of America and of India, and of the sea-routes to the further East and to the Pacific, seem to have roused such questionings as might have been expected. Perhaps, where all was so new, new types of mankind came almost as a matter of course. Certainly, between ecclesiastical authority and colonial prejudice, the status of the lower races settled itself without appeal to ulterior standards. In fact, it was not till what we may call the "Reisejahre" of the eighteenth century that the attention of naturalists was effectively called to the diversities of human types and their significance.

Buffon's two volumes, "Les Variétés Humaines," appeared in 1749, and mark the independent beginnings of the science. The rest of the "Histoire Complète de l'Homme," of which they were the first instalment followed twenty years later, and immediately after the return of Captain Cook from his first circumnavigation. The second edition follows also closely upon the completion of the second voyage (Cook, 1774, Buffon, 1779). It is clear enough that Buffon felt himself in presence of problems which he could not presume to solve, for he contents himself with a purely descriptive treatment, and a geographical order which is evidently provisional. He ranks Bimana and Quadrumana as subdivisions of the Linnæan order Primates, and admits one human species, with a variety of races within it, but attempts no further discussion of the taxonomy. The most elaborate and typical of the special researches suggested by his work is Soemmering's "Mémoire sur les Négres," published in 1785.

It is equally characteristic, meanwhile, of the rather earlier work of Linnæus just alluded to (1735), that it opens up exactly those theoretical questions which Buffon is content to avoid. In the uncompromising inclusion of *Homo sapiens* in the first order of the animal kingdom, a direct challenge is thrown down; for the whole problem of the origin, relationships, and characteristics of man is implicitly involved, and controversies are instituted which have largely determined the course of subsequent research, and which can hardly yet be considered closed, though their relative bearings are now perhaps more justly appreciated. Linnæus admits three species—*Homo sapiens*, *ferus*, and *monstrosus*—with four varieties, white, yellow, black, and red, corresponding with the four great geographical divisions, Europe, Asia, Africa, and America. His main positions were accepted by Buffon (1769), Cuvier (1781), and Blumenbach (1779), and remain the basis of all subsequent work on the subject.

The beginnings of craniology, again, were almost contemporary with Buffon's work, but on the later side; for Daubenton's essay, "Sur la Situation du Trou-occipital dans l'Homme et les Animaux," which is of fundamental importance in its larger bearings, appeared in 1764, and Blumenbach's magnificent "Décades Craniologiques," though their publication did not begin till 1790, represent, of course, a large amount of fruitful work in the preceding years. Blumenbach's interests, however, include much that lies outside craniometry, and his first work, "Les Variétés du Genre Humain," marks a great advance in systematic treatment on the geographical catalogue of Buffon. In this he follows Linnæus' fourfold classification of races, but in his later essay, "De Generis Humain Varietate Nativa," published in 1795, after the voyages of Cook, le Vaillant, and others, he adds a fifth race, the Malay.

to complete a classification which has survived many of those which have succeeded it.

The shortcomings of Blumenbach's craniological work, which was based wholly upon the comparison of the top view of his skulls (*norma verticalis*), were supplied in a remarkable way by the contemporary researches of Camper, "Sur les Différences que Présente le Visage chez les Races Humaines," posthumously published in 1791. Here the lateral view is the standard of comparison, and a new accuracy is attained by the angular measurement of the projection of the maxillary bones. "Camper's angle" is, in fact, the prototype of all exact anthropometric criteria, and though his application of the method is in some ways defective, the credit of its discovery is wholly his. Results of the full face comparison of skulls seem to have been first published by Owen; while the hind-view does not appear even in Prichard's latest work.

With the researches of the last-named, we may close our survey of the foundations of Anthropology. Prichard was born in 1746, and consequently fell largely under the influence of the school of Buffon. His published work dates from 1808, and continues almost without intermission over more than forty years. Without giving up, in outline, the advantages of a geographical treatment, he is one of the first to combine with local and physical criteria the tests afforded by the new studies of language and primitive culture. The last of the pioneers, he is the first to take advantage of the wide field now laid open, and will form a convenient point of departure for the examination of the work of the present century. J. L. M.

